# Do You Speak English or Spanish? Language as a Predictor of Health Outcomes Among Hispanic Adolescents

**DOI:** 10.1007/s10903-025-01676-z

**Published:** 2025-03-14

**Authors:** Alyssa Lozano, Dalton Scott, Carolina Fernandez-Branson, Yannine Estrada, Maya I. Ragavan, Cynthia N. Lebron, Guillermo Prado

**Affiliations:** 1 School of Nursing and Health Studies, University of Miami, 5030 Brunson Drive, Coral Gables, FL 33146 USA; 2 Department of Professional Communication, School of Communication, Writing and the Arts, Metropolitan State University, St Paul, MN 55106 USA; 3 University of Pittsburgh and UPMC Children’s Hospital of Pittsburgh, Pittsburgh, PA 15213 USA

**Keywords:** Acculturation, Language, Hispanic, Adolescence, Drug use, Mental health

## Abstract

Language is our primary tool for communication and a salient component of acculturation status among Hispanic populations. Importantly, language is associated with behavioral health outcomes and can identify and confront health disparities among Hispanic adolescents. The purpose of this study was to evaluate the association between adolescent language identity and drug use and depressive symptoms and examine parent-adolescent communication and parent language identity as mediators and moderators, respectively, of this association. We conducted a secondary data analysis (*N* = 746) of a study evaluating the effectiveness of a family-based intervention to prevent drug use and condomless sex among Hispanic adolescents. We evaluated whether adolescent language identity (i.e., Spanish or English) predicted (1) past 90-day drug use and (2) symptoms of anxiety and depression 30-months post-baseline and whether this relationship was mediated by parent-adolescent communication. We also examined whether the mediational pathway was moderated by parent language identity. English language identity was positively associated with past 90-day drug use and this association was mediated by parent-adolescent communication. The mediational pathway was not moderated by parent language identity. There was not a statistically significant association between English language use and anxiety/depression. However, this association was mediated by parent-adolescent communication. The mediational pathway was not moderated by parent language identity. Hispanic adolescents with an English language identity may have a greater propensity for drug use. Results emphasize the importance of promoting biculturalism and considering parent and adolescent language and communication when developing culturally syntonic interventions for Hispanic adolescents.

## Background

The Hispanic population in the United States (U.S.) continues to rise [1] and has become the minority-majority ethnic group in many U.S. cities [2]. Despite this shifting demographic landscape, there is a dearth of linguistically affirming and culturally tailored research methods and interventions to meet Hispanic behavioral health needs (e.g., drug use, anxiety, depression). This scarcity contributes to the existing behavioral health disparities in drug use and negative mental health outcomes among Hispanic youth when compared to non-Hispanic, Whites [3]. Moreover, since language is our primary means for expression and communication, it is likely that decades of inequitable research and intervention development have also contributed to significant behavioral health differences between Spanish-speaking and English-speaking Hispanics [4]. This is likely due to the fact that non-English speaking (NES) participants are often excluded from research, which may further perpetuate health disparities [5].

### Theoretical Framework

The exclusion of NES participants in research is troubling given the possible impacts of acculturation on behavioral health outcomes among Hispanic populations. Acculturation describes the process of immigrant populations adopting the host culture’s customs and values over time [6]. Existing acculturative frameworks suggest that biculturalism, wherein individuals maintain some customs and values from their heritage culture and adopt some customs and values of the host culture, is indicative of positive psychological adaptation [7]. Language identity and acculturation are intertwined to the extent that language identity has been used as one indicator of acculturation status among Hispanic populations [8, 9]. However, Hispanic parents and their adolescent youth acculturate at different rates and to different degrees, suggesting discord may develop between parents and adolescents that affects their communication quality [10, 11]. For example, adolescents who have lived in the U.S. for the majority of their lives may experience more conflict with their family as they acculturate to U.S. culture (e.g., by preferring to speak English) while their parents - intentionally or unintentionally - maintain ties to their traditional Hispanic cultural values (e.g., preferring to speak Spanish). These discrepancies in language identities, which are proxies for acculturation, may be one factor that contributes to a lack of communication between parents and adolescents. Moreover, language can be a structural barrier for youth such that those youth who use languages other than English (i.e., Spanish) face barriers navigating services which are English centric, and youth are likely embedded in educational systems that do not include education in Spanish.

Importantly, English language use has been associated with poor behavioral health outcomes among Hispanic adolescents. For instance, when Hispanic youth have an English language identity (i.e., those who are more acculturated), they are twice as likely to report smoking cigarettes [9] and significantly more likely to report marijuana and polydrug use than those who prefer to speak Spanish [8]. Hispanic youth who are highly acculturated are also more likely to report alcohol use [12]. Moreover, more acculturated Hispanics are at higher lifetime risk for mental illness [12, 13]. These acculturation differences may contribute to existing behavioral health disparities as parents whose language identity is Spanish, or who cannot speak English, are less able to communicate, and, in turn, effectively engage with their adolescents over behavioral health issues. The challenge of navigating parenting dynamics due to differing acculturation rates between parents and adolescents likely contributes to behavioral health disparities among Hispanic adolescents from different language backgrounds. In addition, it also sheds light on the broader disparities that exist between Hispanics and non-Hispanic Whites, whom are more likely to have spent their developmental years in parent-adolescent language concordant homes. While associations between language use and behavioral health issues have been identified [14], little research attention has been given to the mechanisms through which these associations may exist.

**Fig. 1:**
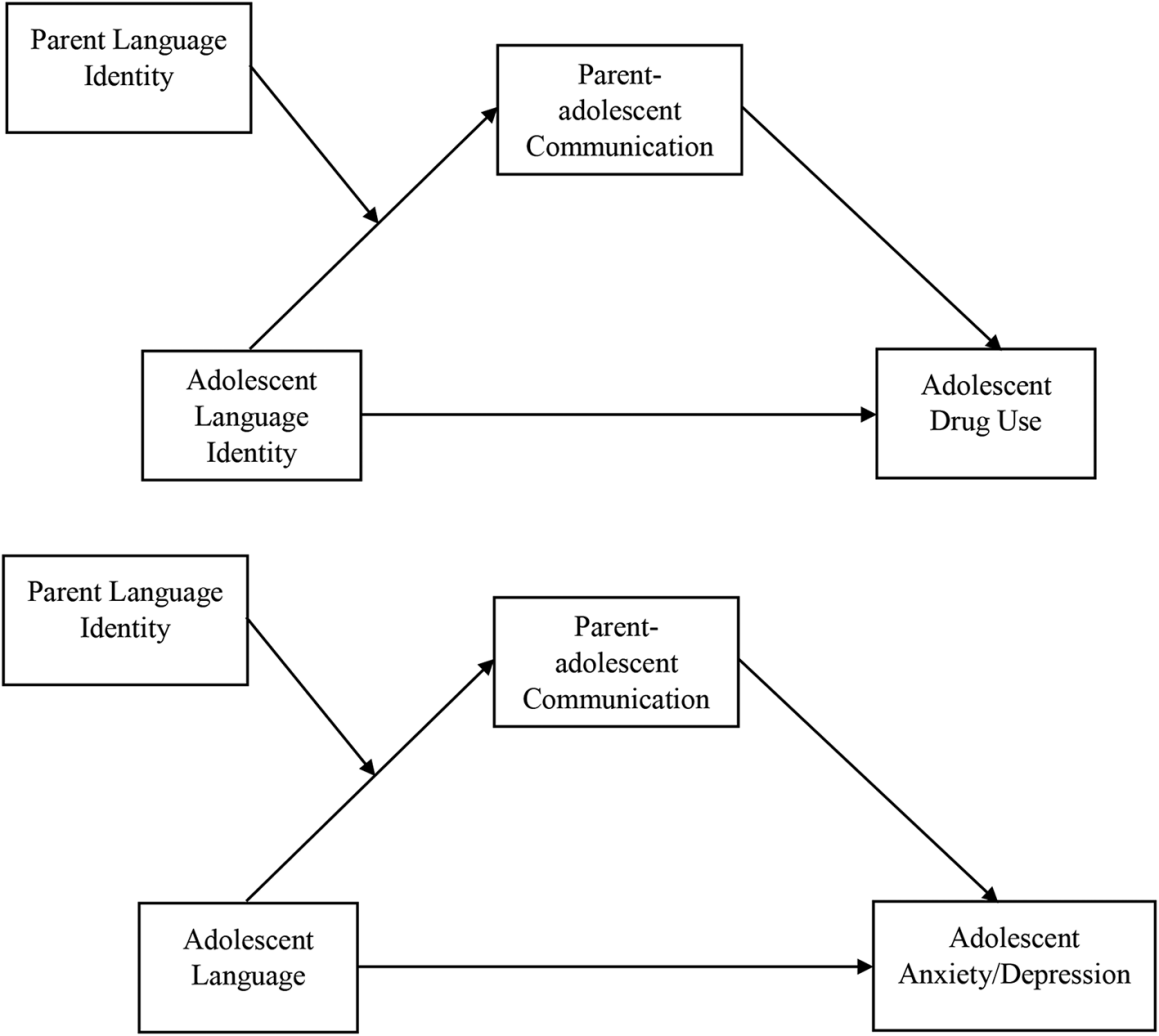
Hypothesized study model

This paper focuses on language identity as a way to begin to disentangle acculturation and its effects on drug use and depressive symptoms. By focusing on language as a key factor in acculturation, prevention efforts can mitigate potentially modifiable risks associated with language barriers while bolstering protective factors tied to linguistic identity and simultaneously ensuring linguistic equitability. Thus, the purpose of this study was to evaluate the association between adolescent language identity and drug use and depressive symptoms and a potential mechanism for this association. We hypothesized that adolescents who prefer to speak in English would be more likely to report drug use and depressive symptoms. Further, we hypothesized that this relationship would be mediated by parent-adolescent communication quality and that the mediation pathway would be moderated by parental language identity (See Fig. 1).

## Methods

### Participants and Data Collection

We conducted a secondary data analysis (*N* = 746) of adolescent-reported data from a randomized controlled trial evaluating the effectiveness of a family-based intervention in preventing drug use and condomless sex among Hispanic adolescents. The intervention consisted of eight parent and family group sessions over the course of three months. The intervention was culturally syntonic, meaning it was designed to be harmonious with the cultural values, beliefs, and practices of the target population (i.e., Hispanics), thereby enhancing acceptance, engagement, and effectiveness [15]. This concept aligns with the broader framework of culturally adaptive interventions, which involves systematically modifying evidence-based treatments to be compatible with the participants’ cultural patterns and values [16]. Intervention facilitators were fluent in Spanish. Group sessions were conducted in Spanish while family sessions were conducted in the family’s language preference. Because 89% of parents preferred Spanish, family sessions were mostly conducted in Spanish. Participants randomized to the control condition did not receive any intervention components. The intervention was effective in reducing drug use frequency and improving family functioning. Further details can be found elsewhere [17]. Briefly, participants were recruited from 18 middle schools in Miami-Dade County, Florida and were eligible to participate if they were of Hispanic origin, in eighth grade, lived with a primary caregiver who was willing to participate, lived within the catchment areas of the participating middle schools, and planned to live in South Florida for the duration of the study. Parents of eligible adolescents and adolescents completed informed consent and assent, respectively, prior to completing the baseline assessment. All study procedures were approved by the University of Miami Institutional Review Board and the Miami-Dade County Public School System.

### Measures

Adolescents answered survey questions related to parent-adolescent communication, past 90-day drug use, and symptoms of anxiety and depression. Adolescent and parent language was determined by the language in which the survey was taken. More than 8 in 10 adolescents (84.2%, *n* = 628) but less than one third of parents (30.8%, *n* = 513) completed the survey in English. All measures were translated and back translated using procedures outlined by bilingual research staff [18].

#### Parent-Adolescent Communication

The Parent-Adolescent Communication Scale (20 items; α = 0.88; [19]) asked adolescents how they communicate with their primary caregiver. For example, “My primary caregiver is always a good listener.” Youth rated each item on a 5-point Likert scale (1 = strongly disagree to 5 = strongly agree). Parent-adolescent communication scores were summed with scores ranging from 20 to 100 with higher scores indicating better communication quality.

#### Drug Use

We adapted items from the Monitoring the Future survey [20] to assess past 90-day frequency of illicit drug use (i.e., marijuana, inhalants, cocaine, LSD, PCP, ecstasy, mushrooms, speed, ice, and heroin) at 30-months post baseline. Past 90-day frequency items for marijuana, inhalants, cocaine, LSD, PCP, ecstasy, mushrooms, speed, ice, and heroin were combined (i.e., endorsements were summed) into one past 90-day drug use variable.

#### Anxiety and Depression

Items from the anxious/depressed subscale of the Youth Self Report (13 items, α = 0.84; [21]) were used to assess symptoms of anxiety and depression among adolescents at 30-months post baseline. Sample items included: “*I cry a lot*.” Responses were rated on a 3-point ordered categorical scale (0 = not true to 2 = very true or often true).

### Analysis

**Fig. 2:**
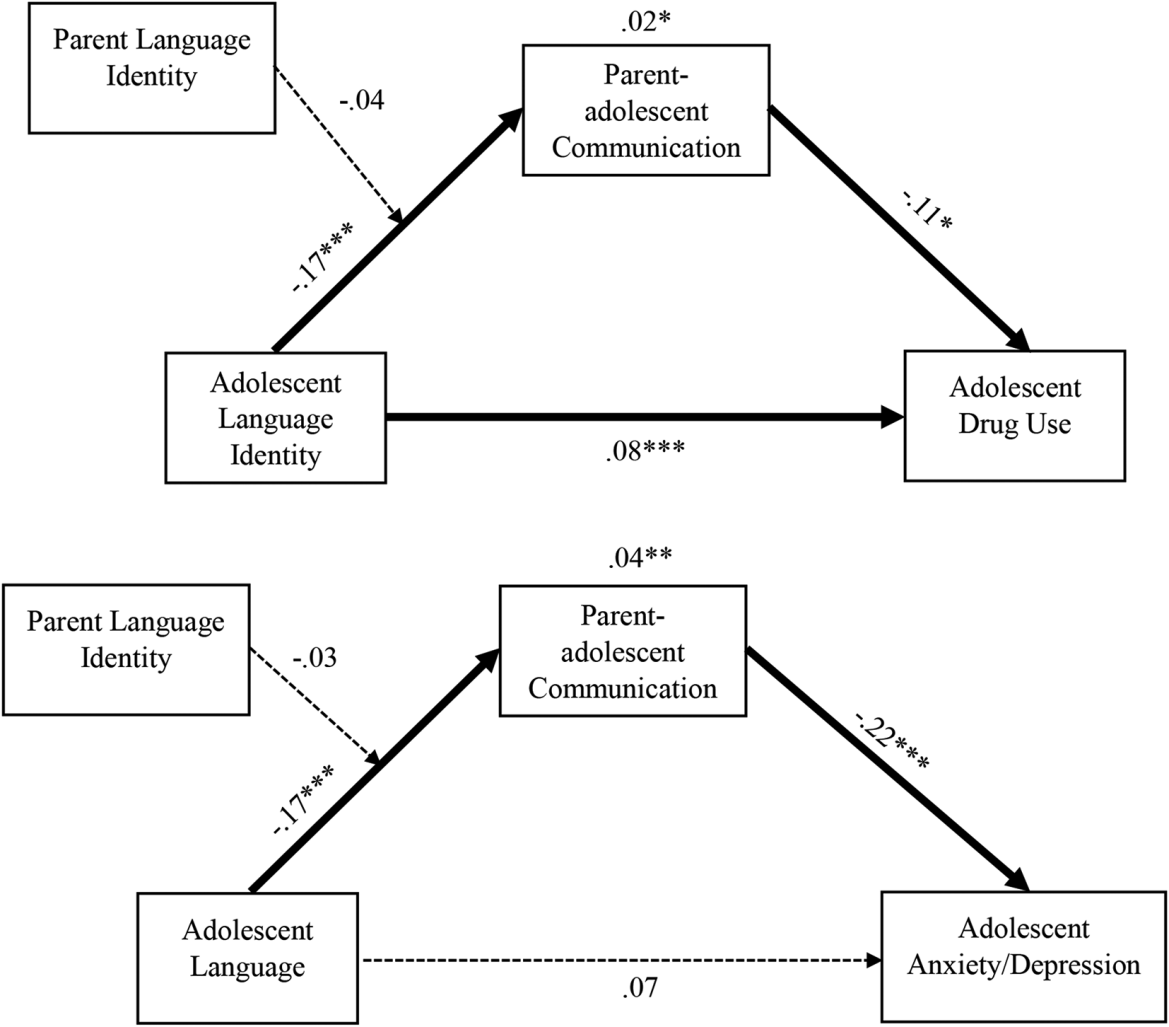
Model results. *Note* The solid paths indicate statistically significant relationships, and the dashed paths indicate non-significant relationships *p <.05, **p <.01, ***p <.001

The analysis consisted of several steps. First, descriptive statistics including means and standard deviations were computed for continuous variables, and frequencies and proportions were calculated for categorical variables. Second, we used structural equation modeling to test our hypotheses. We tested two mediation models for the direct effects of adolescent language on past 90-day drug use 30-months post-baseline, and anxiety and depression 30-months post-baseline with its indirect effect through parent-adolescent communication. Tests of mediation were conducted using the “product of coefficients” test described by MacKinnon [22, 23]. This procedure tests whether the product of the coefficients from language to parent-adolescent communication (a) and from the parent-adolescent communication to the outcome (b; drug use or anxiety/depression) is significantly different from zero. We also examined whether these mediation models were moderated by parent language (i.e., if the indirect effect varied as a function of parent language). To do so, we created an interaction term between adolescent language and parent language to model parent language as a moderator of the relationship between adolescent language and behavioral health outcomes (See Fig. 2).

Adolescent gender, age, nativity, time in U.S., and intervention condition were included as control variables in both models. Model fit was examined using the Comparative Fit Index (CFI) with values > 0.90 indicating good fit, Tucker-Lewis Index (TLI) with values > 0.90 indicating good fit, Root Mean Square Error of Approximation (RMSEA) with values < 0.08 indicating good fit, and Standardized Root Mean Squared Residual (SRMR) with values < 0.05 indicating good fit [24, 25].

## Results

The majority of adolescents were Male (52.1%), born in the United States (54.8%) and identified their language identity as English (84.2%). Conversely, a majority of parents reported Spanish language identity (69%). Full participant demographics can be found in Table 1.

**Table 1:** Participant demographics

Variable	M (SD) or *N* (%)
Adolescent	
** Age (12–16)**	13.38 (0.68)
** Gender**	
Male	389 (52.1)
Female	357 (47.9)
** Country of Origin**	
U.S. Born	409 (54.8)
Foreign Born	335 (44.9)
** Time in US**	
Less than 3 years	110 (14.8)
Between 3 and 9 years	142 (19.1)
More than 9 years	492 (66.1)
** Language Identity**	
English	628 (84.2)
Spanish	116 (15.5)
** Parent-adolescent Communication (20–100)**	69.92 (14.81)
Spanish Concordance (52–100)	76.56 (11.84)
English Concordance (22–100)	68.10 (15.44)
Parent Spanish and Adolescent English (20–98)	69.32 (14.70)
** Past 90-day Drug Use (0–1)**	0.05 (0.22)
Less than 3 years	0.03 (0.18)
Between 3 and 9 years	0.04 (0.20)
More than 9 years	0.06 (0.24)
** Depression and Anxiety Symptoms (0–24)**	4.60 (3.00)
Less than 3 years	4.32 (4.62)
Between 3 and 9 years	4.13 (4.05)
More than 9 years	4.80 (4.59)
***Parent***	
** Language Identity**	
English	230 (31.0)
Spanish	513 (69.0)

Note. The past 90-day drug use variable measures the frequency (i.e., number of times) of use over the past 90 days and is represented as a mean. The measure of depression and anxiety provides information on the possible presence of depressive symptoms but that it is not a diagnostic tool

### Drug Use

In the drug use model, the model fit for the entire sample was acceptable: χ2 (12) = 29.72, *p* <.01; RMSEA = 0.05 (90% CI = [0.025, 0.065]), CFI = 0.98, TLI = 0.96, SRMR = 0.03. English language was positively associated with past 90-day drug use 30-months post-baseline (*b* = 0.08, *p* <.001) and negatively associated with parent-adolescent communication quality (*b* = − 0.17, *p* <.001). The direct effect was mediated by parent-adolescent communication (*b* = 0.02, *p* <.05), but the mediational pathway was not moderated by parent language (*b* = − 0.04, *p* =.40).

### Anxiety and Depression

Model fit for the anxiety and depression models was also acceptable: χ2 (12) = 52.04, *p* <.001; RMSEA = 0.07 (90% CI = [0.049, 0.086]), CFI = 0.94, TLI = 0.90, SRMR = 0.05. There was a negative association between English language and parent-adolescent communication quality (*b* = − 0.17, *p* <.001), but there was not a significant association between English language and anxiety/depression 30-months post-baseline (*b* = 0.07, *p* =.065). However, this association was mediated by parent-adolescent communication (*b* = 0.04, *p* <.01), but the mediational pathway was not moderated by parent language (*b* = − 0.03, *p* =.37).

## Discussion

The purpose of this study was to (1) evaluate the association between adolescent language identity and drug use and depressive symptoms, (2) examine whether this association was mediated by adolescent-reported parent-adolescent communication quality, and (3) if the mediational pathway was moderated by parent language. We found that English language use among adolescents was positively associated with drug use at 30-months post-baseline and that this relationship was mediated by parent-adolescent communication such that drug use was influenced by how adolescents communicated with their parents, with more positive communication associated with less drug use and more negative communication associated with increased drug use. However, we did not find evidence that this relationship was moderated by parent language. We did not find a significant association between English language and anxiety/depression 30-months post-baseline, but we did find that this association was also mediated by parent-adolescent communication so that positive communication between parents and adolescents was associated with less anxiety/depressive symptoms among adolescents and poor communication was associated with more anxiety/depressive symptoms among adolescents. Like the drug use model, the mediational pathway was not moderated by parent language.

Consistent with prior literature on Hispanic adolescents, we found that English language was positively associated with adolescent drug use [8, 9, 26]. Contributing to the extant literature, we found that parent-adolescent communication mediated the relationship between language identity and drug use which may suggest that adolescent preferences for speaking English may be positively associated to drug use due to the language barrier that may inhibit positive parent-adolescent communication. In this case, the relationship between adolescent English language identity and parent-adolescent communication was negative. If there is poor foundational communication between parents and adolescents, it is unlikely that other critical components of family functioning, such as parental monitoring of peers and discussing behavioral health, will be strong. However, because there were no moderation effects and the relationship between adolescent language identity and parent-adolescent communication did not vary as a function of parent language identity, it may not be the non-concordance in parent-adolescent language, but rather other processes related to adolescent’s acculturation that impact parent-adolescent communication. Moreover, the majority of parents (69%) spoke Spanish, which may have potentially obscured possible moderation effects. Alternatively, families may be bilingual, thus effects may have also been obscured due to the fact that in certain settings (i.e., in homes) there is language concordance among parents and adolescents.

In the context of the study’s findings, the need to improve parent-adolescent communication quality is highlighted by its mediation effect in the relationship between language identity and drug use. This finding suggests additional research is needed on the associations between language identity and parent-adolescent communication quality. Poor parent-adolescent communication quality may subsequently inhibit other components of the parent-adolescent relationship such as effective parental monitoring. Given that adolescence is a time wherein the influence of peers becomes increasingly important, it is likely that the impact of language identity spills over to adolescents’ peer world therefore affecting parental monitoring. Moreover, Hispanic adolescents who are not native English speakers may become more peer-oriented as they gain English language fluency and consequently spend time with other English-speaking adolescents who may be more acculturated and therefore more likely to use drugs [9, 27].

We did not find evidence of a direct relationship between English language use among adolescents and anxiety/depression symptoms. Despite the stigma surrounding mental health among Hispanic populations, recognition of mental illness (including symptoms, signs, and modes of expression) may lead to help-seeking among Hispanic parents. In a study by Dixon De Silva et al., although Hispanic parents endorsed stigma about mental health and seeking treatment for mental health, parents indicated that they wanted to help their children improve their symptoms of depression [28]. This is likely due to the fact that Hispanic parents may focus on outwardly visible behaviors as opposed to moods. In the same study, parents noted that they became aware of their adolescent’s mental health challenges by observing symptoms such as adolescent’s isolating behaviors or not talking [28]. It may be that once youth begin to have visible signs and symptoms of anxiety and depression, parents may feel as though there is an urgency to address these signs and symptoms despite pre-conceived notions of mental health [29–31]. Similar to the drug use model, we found that parent-adolescent communication mediated the relationship between adolescent language use and symptoms of anxiety/depression, highlighting the protective effect of parent-adolescent communication. Therefore, this study elucidated the need to improve communication between Hispanic parents and adolescents as a way to improve behavioral health outcomes.

### Implications

This paper focuses on one aspect of acculturation as a way to begin to disentangle this construct and its effects on drug use and depressive symptoms. This study’s use of language identity as a proxy for acculturation demonstrates that greater acculturation to the U.S. may adversely impact outcomes related to drug use. Results emphasize the importance of considering both parent and adolescent language use when developing and enhancing culturally syntonic interventions for Hispanic families and promoting biculturalism (i.e., both Hispanicism and Americanism) in Hispanic homes to prevent poor health outcomes among Hispanic adolescents. Family-based interventions for Hispanics that focus on improving family relationships should pay special attention to how parents and adolescents navigate language differences, an important indicator of acculturation, when communicating.

English-language use among adolescents is one proxy variable for acculturation, and this work supports the literature that suggests youth who are more acculturated are more likely to use illicit substances. Thus, proxy variables for acculturation, such as language use, and understanding how they relate to substance use and mental health is an important step in understanding if culturally tailored and linguistically equitable interventions in the home with parents and adolescents can reduce substance use. Our findings also suggest that communication between adolescents and parents is a mediator of substance use. Therefore, fostering positive communication skills between parents and adolescents potentially discordant language identities is important in behavioral health interventions aimed at reducing substance use among Hispanic youth.

In addition to considerations around language identity and parent-adolescent communication in the development of culturally tailored and linguistically equitable interventions, it is also important to consider how to ameliorate structural barriers around language. Oftentimes, non-English-speaking parents and adolescents are embedded in systems that are English centric. This may lead to barriers in navigating a variety of services. Such structural inequities may present a challenge to retaining native languages (i.e., Spanish) which may then subsequently perpetuate health disparities related to various health outcomes. Research focused on health disparities among non-English-speaking individuals should thus promote language justice which is centered on the promotion of the right to communicate in individuals’ languages to better address power imbalances and achieve equity.

### Limitations and Strengths

This study has some limitations that should be acknowledged. First, by using survey language, we assume that preference of language in the survey translates to language preferences in other aspects of life. This does not account for youth who may use both English and Spanish (i.e., are bilingual). Future research should evaluate additional details related to youths’ language preferences in different settings and with different groups (i.e., peers versus parents). Relatedly, neither acculturation or specific culture-related variables were measured in this study, which may provide an understanding of how cultural orientation may impact health outcomes. However, as noted, language is one aspect of acculturation that can provide insight to develop culturally syntonic interventions that are linguistically equitable and ameliorate structural barriers around language. Second, data were collected from participants in one geographical area in South Florida, which may not be generalizable to other Hispanics, nationally. However, there were several countries in Central and Latin America represented in our sample. Third, data for this study was self-reported by adolescents, and drug use and anxiety/depression may have been underreported or overreported. Finally, we measured mental health with a subscale from a larger measure, however, it has been used in other studies with Hispanic adolescents [32, 33].

### New Contribution to the Literature

Despite these limitations, this study has several strengths that should be highlighted. First, we examined how adolescent language, an important proxy for acculturation, can impact adolescent health outcomes related to drug use and mental health over time. Moreover, given the saliency of family for Hispanics, we were also able to examine how these relationships may be impacted by parent-adolescent communication and parent language; thus, highlighting the importance of potential family-based communication interventions for improving Hispanic adolescent behavioral health outcomes. The findings of this study can inform the development and refinement of culturally syntonic interventions for Hispanic families to begin to ameliorate health disparities related to drug use and mental health among Hispanic adolescents.

## Data Availability

The datasets generated during and/or analyzed during the current study are available from the corresponding author on reasonable request.
